# Cumulative cultural evolution, population structure and the origin of combinatoriality in human language

**DOI:** 10.1098/rstb.2020.0319

**Published:** 2022-01-31

**Authors:** Simon Kirby, Monica Tamariz

**Affiliations:** ^1^ Centre for Language Evolution, University of Edinburgh, Edinburgh, UK; ^2^ Department of Psychology, Heriot-Watt University, Edinburgh, UK

**Keywords:** combinatoriality, population dynamics, cultural evolution of language

## Abstract

Language is the primary repository and mediator of human collective knowledge. A central question for evolutionary linguistics is the origin of the combinatorial structure of language (sometimes referred to as duality of patterning), one of language’s basic design features. Emerging sign languages provide a promising arena to study the emergence of language properties. Many, but not all such sign languages exhibit combinatoriality, which generates testable hypotheses about its source. We hypothesize that combinatoriality is the inevitable result of learning biases in cultural transmission, and that population structure explains differences across languages. We construct an agent-based model with population turnover. Bayesian learning agents with a prior preference for compressible languages (modelling a pressure for language learnability) communicate in pairs under pressure to reduce ambiguity. We include two transmission conditions: agents learn the language either from the oldest agent or from an agent in the middle of their lifespan. Results suggest that (1) combinatoriality emerges during iterated cultural transmission under concurrent pressures for simplicity and expressivity and (2) population dynamics affect the rate of evolution, which is faster when agents learn from other learners than when they learn from old individuals. This may explain its absence in some emerging sign languages. We discuss the consequences of this finding for cultural evolution, highlighting the interplay of population-level, functional and cognitive factors.

This article is part of a discussion meeting issue ‘The emergence of collective knowledge and cumulative culture in animals, humans and machines’.

## 1. Introduction

Humans use language daily throughout their lives. Language is not only a key repository but also the primary mediator of collective knowledge in our species. Investigating the mechanisms by which the properties of linguistic structure that enable these functions emerge and evolve will contribute to a more comprehensive understanding of cultural transmission and cumulative evolution. Like all cultural traits, linguistic structure is the result of evolutionary processes, and that includes adaptation to multiple pressures [1–3]. The Linguistic Niche Hypothesis [4] proposes that linguistic structure reflects pressures derived from properties of the population in which cultural evolution takes place, including population size, social network structure and patterns of interaction dynamics. In addition, the iterated learning literature [5–9] highlights the role of individual cognitive biases and preferences unfolding over cultural transmission on the evolution of linguistic structure. The present study explores the evolution of combinatorial structure in language as an adaptation to learning biases, and the effects of population dynamics on this evolutionary process.

### (a) Social and demographic factors

Cultural evolution is influenced by population size and structure [10–13]. In particular, the size and structure of social networks have been suggested to modulate the balance between two mechanisms of evolution: neutral drift and selection in the cultural evolution of language [14–17]. Additionally, temporal connectivity dynamics, or the detailed sequence of interactions among individuals, also affect evolution. When sub-groups within the population remain relatively isolated from each other for a period of time, the evolution by selection of adaptive cultural variants in the population slows down, reflecting an increased influence of neutral cultural evolution, or drift [18]. If population size is kept constant, stronger selection pressures (or, conversely, weaker drift effects) accelerate evolution. And the effects of drift are felt more strongly in smaller populations [19]. Therefore, differences in the distribution of cultural traits in populations of different sizes and structures may ultimately be the outcomes of evolution driven by different balances between selection and drift, which in turn influence the *rates* of evolution.

Population size and contact in particular have been shown to affect linguistic structure. Languages used by larger communities, covering larger geographical areas with a higher degree of contact with other languages, tend to be simpler than those used in smaller, more close-knit populations [4,20–30]. This has been suggested to derive from the presence of adult, non-native language learners [4,31]. Adult learners simplify their input by making it more regular. They find it easier to learn more regular languages [32], and when they learn a second language, they have particular problems learning complex patterns such as morphological agreement, noun case, number and gender, or verbal tense [21,33–37], even when they have analogous complex structures in their first language [38]. Changes introduced by adult learners may spread to others in the population. When native language users have frequent interactions with non-natives, they have a higher probability of adopting [23,39,40] and amplifying [41] simplified forms, and also of producing simpler ‘foreigner directed speech’ [31,42].

Early in development, children also show a preference for simpler, rule-based items over exceptional or irregular ones in language [43]. As they develop, they gradually learn and adopt the complexities in their culture and language. This indicates that all learners initially tend to simplify their linguistic input. Importantly, this has different consequences depending on whether the learners are adults or children. While children’s short-lived simplifications do not normally spread to the population, adults, who seldom reach native-like proficiency, introduce simpler structures during their interactions with native language users. We will return to this point later as we discuss the unusual cases where children, as learners, do contribute to the spread of linguistic structure in a population.

### (b) Combinatoriality

In this paper, we want to demonstrate that these social and demographic factors can interact with cognitive biases to explain the origins of basic design features of human language. Arguably the most fundamental of these design features is what Hockett [44] called *Duality of Patterning* [45,46]. Typically, human languages construct meaningful signals by combining elements at two levels of structure. At the top level, there are meaningful elements (words like *cat* and morphemes like the plural marker *-s*) that can recombine in different ways to construct phrases and sentences whose meaning is *composed* of the meanings of these parts. Thus, *compositionality* is a key property of language. These meaningful elements are further constructed out of combinations of meaningless elements—the phonemes such as */t/*. Thus, *combinatoriality* is another key property of language.^1^ There is nothing about the */h/* in *hat* or the */k/* in *cat* that carries the meaning distinction between these two words. Nor is there anything about the rhyme */æt/* that somehow conveys what is similar between *cat* and *hat*. Pairs of words like cat/hat are called *minimal pairs* because a meaning distinction is induced by making a minimal change to the combination of meaningless elements. If a communication system has examples of minimal pairs, then we have very clear grounds for saying it exhibits duality of patterning, although strictly speaking, minimal pairs are not a necessary consequence of having a combinatorial system.

The existence of combinatorial structure is not restricted to one modality of language. Sandler *et al.* [47] give examples of minimal pairs in Israeli Sign Language (ISL), which illustrate how a lexicon of meaningful signs can be created out of recombinations of a small set of meaningless elements. While in spoken language phonemes recombine, the minimal (meaningless) elements in ISL relate to four features of the dominant hand in a sign: movement, handshape, orientation or location.

Organizing a communication system in this way, with two layers of recombination—one meaningless, and one meaningful—seems like an elegant design. Nevertheless, duality of patterning is *not* inevitable. There is remarkably little evidence for it in non-human communication [48] (though see [49–52] for some exciting counterexamples), and it is absent from at least one human language, Al-Sayyid Bedouin Sign Language (ABSL). In contrast to ISL, there is no evidence of minimal pairs in the lexicon of ABSL [47]. Each sign in ABSL appears to be built not from a recombination of phonological elements that robustly reoccur across a wide set of distinct lexical items, but rather as a non-decomposable whole, demonstrating that duality of patterning is not an inescapable consequence of the existence of a communication system.

The fact that combinatoriality is so widespread, but not obligatory, poses two interesting challenges for evolutionary linguistics: what are the origins of combinatoriality? And why does it sometimes not appear? In the next section, we set out our hypotheses for answering these questions, before turning to a computational model testing these hypotheses in §3.

## 2. Hypotheses

Of particular relevance here is work that aims to observe the emergence of combinatoriality in laboratory experiments [53–58]. For example, in Verhoef’s transmission chain experiments, participants first listen to a set of signals made using a continuously variable pitch slider (e.g. a slide whistle) and then they have to reproduce these signals. The signals that subsequent participants are asked to reproduce are the signals that the previous participant in the chain produced. The initial sets of signals that participants copy are not combinatorial. However, over episodes of transmission, discrete sub-parts of the signals start to be reused across multiple signals. This process of *iterated learning* of sets of signals is sufficient to lead to the gradual emergence combinatorial structure.

This experimental work suggests that cultural evolution is a candidate explanatory mechanism for the origins of duality of patterning, and specifically of combinatoriality, in language. Models of the cultural evolution of linguistic structure have identified cultural evolution as the engine behind the emergence of linguistic structure as an optimal trade-off between two partially competing pressures—learning and communication [9]. For example, Kirby *et al.* [59] used transmission chain experiments and computer simulations to demonstrate that compositional structure arises in language only when there is both a pressure for a language to be learnable by multiple subsequent generations of learners, and for it to be used for communicating distinctions between different meanings.

To understand why, it is worth considering first what the pressures would be on a language that only needs to be learned by subsequent generations of individuals. The simulation in [59] models learning as a process of Bayesian inference in which hypotheses that are simpler have a higher prior probability. Specifically, languages that can be encoded with shorter descriptions are more learnable. The simplest possible language is one in which utterance is identical (you can imagine a language in which there is just one ‘word’ for every possible meaning). Obviously, this language is very easy to learn. Indeed, in simulations of iterated learning, in which the language of each generation of individuals is acquired by simply observing utterances produced by the previous generation, this *degenerate* language is exactly what emerges. This is also true in the experiment laboratory. Degenerate languages where multiple meanings are expressed using a single signal quickly emerge in transmission chain experiments with miniature artificial languages [8].

Obviously, while they are learnable, degenerate languages are dysfunctional as they cannot be used to communicate distinctions between meanings. To address this, Kirby *et al.* [59] introduce a model of communication [60] in which the simulated agents prefer to produce signals that would discriminate between distinct meanings (on the assumption that their interlocutor has the same language as themselves). In a model (and laboratory experiment) in which two such agents take it in turns to communicate, languages emerge that are communicatively effective in that they can be used to discriminate meanings. However, they are typically *holistic*, with each meaning being conveyed by a distinct signal. For example, such a language might call a blue square a *foo*, a red square a *bar*, and so on. These languages are good for communication, but are hard to learn because learners can’t generalize from signals they have seen to express unseen meanings. This isn’t a problem for the case of just two individuals communicating with each other because their shared history means that they have less need to generalize.

When both this communicative pressure *and* the repeated transmission to new learners are combined in the model (and laboratory experiments) then *compositional* languages evolve [59]. These are ones in which different aspects of the meanings being conveyed are encoded as different sub-parts of the signals. Like all languages, English is compositional in this sense. We know that *blue square* refers to a blue square because we know the meaning of *blue* and the meaning of *square* and how these words combine. Compositional languages solve the problem of being learnable and being communicatively functional. They are both simple (in the sense of not requiring too long a description length) and expressive (in the sense of being able to make distinctions between meanings).

This trade-off between simplicity and expressivity (which in some papers is referred to as informativeness) has recently been shown to explain a diverse range of facts about human language [61–66]. The languages that we see in the world exist at the optimal frontier in a space of logically possible languages of varying complexity and informativeness. Crucially, it is cultural evolution by iterated learning among individuals who are trying to communicate rationally that moves languages to this frontier. Given this prior work, we propose the following hypothesis:

**The trade-off hypothesis**. Combinatoriality is the result of cultural evolution trading-off between a pressure from learning leading to simpler lexica, and a pressure from communication leading to expressive ones.

To test this, in the next section, we modify the model of [59]. Whereas in that model meanings were complex (in that they had distinct parts) but signals were relatively simple, here we consider the case where meanings are atomic but signals have greater potential complexity. This allows us to explore a space of languages that vary in their degree of combinatoriality of signals. Specifically, there will be languages in this space with and without minimal pairs. We will use this model to understand the precise conditions under which duality of patterning evolves.

However, the fact that ABSL does not display combinatoriality leads us to wonder what determines whether a language displays this feature. One of the most exciting features of signed languages from the perspective of research in language evolution is that they are all relatively young. Some are younger than others—so much so that we actually in some cases have clear evidence of the process of *emergence*—and it is reasonable to suggest that some design features have yet to appear in these. Emerging sign languages therefore act as real-world equivalents of the processes that we are trying to recreate in laboratory experiments, but with the advantage that the languages are fully-blown natural systems rather than miniature laboratory equivalents. In addition, a common criticism of laboratory experiments is that the participants are all already speakers of a language, which further highlights the value of observing language emergence in the real world. ABSL is just such an emerging sign language, so, one hypothesis might simply be that ABSL hasn’t had the time required to move from its initial holistic starting point to a combinatorial one. Perhaps the pressure from learnability, which arises from a fundamentally *cumulative* process of cultural evolution, will eventually lead to the gradual structuring of the ABSL lexicon. But this only shifts the question on to why other equally young sign languages *do* exhibit duality of patterning. ISL, for example, is approximately the same age as ABSL but from the point of view of the structure within the lexicon it is strikingly different [47].

It is instructive to look at the distinct processes that formed ABSL and ISL for clues as to why there might be this difference. Meir *et al.* [67] talk about the difference between two distinct scenarios for language emergence: village sign and deaf community sign.

A village sign language arises in an existing, relatively insular community into which a number of deaf children are born. A deaf community sign language, on the other hand, arises when a group of deaf individuals, often from different places are brought together (frequently for educational purposes, as in a residential school) and form a community. [67]

ABSL is an example of a village sign language, whereas ISL is a deaf community sign language. We unfortunately lack the quantitative data on historical social network structure in these two language emergence scenarios that we would need to make definitive statements about the cultural evolutionary processes involved. However, we feel it is reasonable to suggest that in the deaf community scenario there will be a more substantial role for horizontal transmission (via peer-to-peer learning) of the emerging language, whereas in the village sign language scenario there will be a more substantial role for vertical transmission (e.g. learning the sign language from older generations within families).

This potential difference between these two types of sign language emergence scenarios suggests the following hypothesis:

**The learning from learners hypothesis**. In deaf community sign languages, the school context promotes learning from individuals who are also learners, and this accelerates the cultural evolution of structure in response to learning-based biases.

In order to test the learning from learners hypothesis, we implement two different population structures in our simulation. In the village sign language scenario, agents always learn from the oldest agent in the population. By contrast, in the deaf community setting, agents learn from a producer who is half way through their lifespan, and therefore still in the process of language learning.

## 3. Simulation model

In this section, we set out a relatively small modification to the model from Kirby *et al.* [59] with simple rather than complex meanings that enables us to test our two hypotheses.^2^

### (a) Language model

As in [59], we seek the absolute minimal model for this investigation. In other words, we want to find the smallest possible space of languages in the model that nevertheless exhibits the possibility for combinatorial and non-combinatorial outcomes. For this purpose, we will consider languages made up of a lexicon of four signals, conveying four completely distinct atomic meanings. Every language in our space of possible languages will have one and only one signal for each of these four meanings. However, more than one meaning may share the same signal.

The signals will be drawn from a set of eight possible combinations of two letters: ac, bd, ad, bc, pr, ps, qr, qs. We can think of the first and second letter in these signals as corresponding to an abstract representation of a phonological feature (for example, onset and rhyme in a syllable, or location and handshape in a sign). Note that in this tiny set of possibilities, each of these features can be ‘filled’ with a distinct set of values: a, b, p, q, for the first letter, and c, d, r, s, for the second. Furthermore, there are some constraints on possible combinations: a & b only occur with c & d, and p & q only occur with r & s.

Given that there are eight possible signals and four meanings, there are 4096 possible languages in total. This set of signals allows for several important sub-types of language in this space (we give examples of each as an ordered list of signals, one for each of the four meanings):

**Degenerate** languages in which every meaning is expressed with the same signal. There are eight of these, including: ac ac ac ac

**Holistic** languages in which every meaning is expressed with a distinct signal, but there is no reuse of parts of the signals across different meanings. There are 1632 of these, including: ac bd pr qs

**Combinatorial** languages in which there is maximum reuse of parts of signals without introducing ambiguity by collapsing distinctions across any pair of meanings. There are 48 of these, including: ac ad bc bd

Of these three types, only the last contains minimal pairs and exhibits duality of patterning. For the graphs, we will also plot ‘other’ language types, which do not fit into one of these three categories. For example, the partially degenerate language, ac ac pr qs falls into this category.

Now clearly, this model of language is massively simplified. However, it is rich enough to exemplify the phenomenon we are interested in and allow for the potential for a range of outcomes. In addition, it is simple enough that, as we shall see in the following sections, we can run simulations of populations of interacting learners over multiple generations and compute the posterior probabilities over all languages without resorting to approximations.

### (b) Bayesian learning model

We used a Bayesian model of learning to simulate individual agents. In this approach, learners compute a posterior probability distribution over all hypotheses (which in this case are the 4096 different languages) by combining data that they have seen with a prior distribution over those hypotheses: *P*(*l*|*d*) ∝ *P*(*l*)*P*(*d*|*l*), where *P*(*l*) is the prior probability of language *l*, and *P*(*d*|*l*) is the likelihood of seeing the data *d* given the language *l*.

It is the prior distribution that captures the preference for simpler hypotheses. So, we need to ask here what makes a simple or complex language in this space? Clearly, the degenerate languages are simplest since they use the same signal for every meaning. Knowing one of these languages amounts to just knowing a single word. It also makes intuitive sense that the holistic languages are maximally complex because every meaning is expressed with a completely distinct signal. So, the combinatorial languages exist somewhere between these two. It turns out that there is a very natural way to capture these intuitions based on the coding length of the languages in bits [68,69]. The coding length of a language is given by

$$L(l)=-\sum \log_{2}\;p(l_{i}),$$

where *p*(*l*_*i*_) is the probability of the *i*th character of the language.^3^

Each of the three types of language have a different coding length under this scheme given below (in order of increasing complexity). Note that the log probability here is multiplied by 8 in each case because there are eight characters in total in the representation of the language.

**Degenerate** language, ac ac ac ac. $L(l)=-8\times \log_{2}\frac{4}{8}=8\;\mathrm{bits}$
**Combinatorial** language, ac ad bc bd. $L(l)=-8\times \log_{2}\frac{2}{8}=$ 16 bits
**Holistic** language, ac bd pr qs. $L(l)=-8\times \log_{2}\frac{1}{8}=24\;\mathrm{bits}$

We can transform this code length in bits into a prior probability distribution in such a way that more complex languages have a lower prior probability: *P*(*l*) ∝ 2^−*L*(*l*)^

In addition to the prior probability of each language, in order to compute the posterior probability of each language in the space, learners need to be able to compute the likelihood of the data seen given that language. In this case, the data are a set of meaning–signal pairs produced by other agents. In this simulation, learners make a simplifying assumption that the data they see are the product of a single language and that the probability of a particular meaning–signal pair is close to 1 if and only if that signal is paired with that meaning in the language and close to 0 otherwise

$$P(s|l,m)\propto \left\{ \begin{array}{cc} 1-\epsilon & \mathrm{if}\;s\;\mathrm{paired}\;\mathrm{with}\;m\;\mathrm{in}\;l\\ \frac{\epsilon }{3} & \mathrm{otherwise} \end{array}\right.,$$

where *s* is the signal, *m* is the meaning and *l* is the language. *ε* is an error term that represents the probability that a sender will mistakenly send one of the three incorrect signals for the meaning. In the simulations reported here, *ε* = 0.05.

Together, the likelihood and prior terms allow agents to compute the posterior probability of each of the 4096 languages in the hypothesis space given the full set of meaning–signal pairs they have observed. This posterior is updated every time the agent observes a new meaning–signal pair. This complete posterior distribution then is used as input to the communication process, which we describe next.

### (c) Communication

The model of communication we use is inspired by the Rational Speech Act framework [60], albeit simplified for our purposes. Consider the case where an agent has to produce a signal for a particular meaning. A rational agent would take into account the posterior distribution over possible languages in order to produce a signal that is most likely to be associated with the intended meaning across all the languages, weighted by their posterior probability. However, this does not take into account the fact that a receiver of a signal does not know what the meaning is, so even if the correct meaning is associated with that signal in a particular language, if there are other meanings in that language with which that signal is also associated, then there is the possibility of a failure of communication. To take that into account, we introduce a communicative rationality parameter *γ*, which determines how much an agent considers the risk of ambiguity for each particular signal and language.

This gives us a ‘communication’ function, which gives a communicative weighting for each signal given a language and meaning *C*(*s*|*l*, *m*):

$$C(s|l,m)=\left\{ \begin{array}{cc} {\left(\frac{1}{a}\right)}^{\gamma } & \mathrm{if}\;s\;\mathrm{paired}\;\mathrm{with}\;m\;\mathrm{in}\;l\\ 0 & \mathrm{otherwise} \end{array}\right.,$$

where *a* is the ambiguity of a signal in a particular language, which is simply the number of meanings in that language with which the signal is associated.

This equation combined with the posterior probability distribution gives us our complete model of signal production. Signallers pick the signal to produce, *s*_*P*_, that maximizes the product of the sum of the weighted posterior probabilities of the languages where that signal is associated with the correct meaning:

$$s_{P}={\mathrm{argmax}}_{s\in S}\sum_{l\in L}P(l|d)C(s|l,m),$$

where *L* is the set of all possible languages and *S* is the set of possible signals.^4^

If *γ* = 0, then agents only care that the target meaning is one of the possible interpretations of the signal for a given language. If *γ* ≫ 0, then agents increasingly avoid using signals that could potentially lead to incorrect interpretations. In this way, we can essentially dial up or down the pressure from communication in our simulations to see how this affects the evolution of combinatorial structure in the language. After *s*_*P*_ is found, then the signaller produces that signal, with a probability 1 − *ε* and a random other signal otherwise, to simulate errors in production.

### (d) Population model

Now that we have the structure of an individual agent in the model in place, we turn to how they interact and learn from each other in a population. There are many ways this can be set up, but for the results reported here we use a population of 10 agents, all of whom are initialized at the start of the simulation to using the same holistic language. We feel this is a reasonable starting point because it is a language that is maximally distant from a combinatorial one while still being optimal for communication. We also know from the history of emerging sign languages such as ABSL that a starting point that is communicatively effective but that does not yet exhibit systematic combinatorial structure is at least plausible.

To model the village sign language setting, with vertical rather than horizontal transmission, the simulation then iterates over the following steps:

1. The oldest agent is removed from the population.
2. A new untrained agent is added to the population.
3. The following communication/learning steps take place 20 times:
  (a) A meaning is chosen at random.
  (b) The oldest agent produces a signal for that meaning.
  (c) A different, randomly chosen agent from the population observes the meaning–signal pair and updates its posterior.

Note that in this set-up, each agent will potentially be exposed to data from nine other agents in their lifetime, and it is always the most experienced agent who provides data for less experienced agents (we alter this feature of the simulation in a later section).

To implement the deaf community or school setting (learning from learners condition), we make only one modification. We change which of the agents is producing signals when a pair of agents is chosen for communication. Whereas in the previous condition, the oldest agent was always chosen, in these runs, we choose the producer to be one who is still in the process of language learning. In the previous condition, across the whole of their lifetime, an agent will learn from nine other agents, all of whom will be older than them. In this simulation, across the whole of their lifetime, an agent will still learn from nine other agents. However, this time five of those agents will be older than them and four will be younger.

Obviously, this is not a particularly realistic model of a school context where learners are embedded in a rich social network of interactions. It would be relatively straightforward to model this richer kind of social network structure in a simulation like this, but in the spirit of keeping things as simple as possible, we decided to make only this one minimal change from the previous simulation. Specifically, the population size, communicative rationality, prior bias, length of lifespan, number of different individuals encountered in that lifespan, and age at which we measure the data, are all kept identical as in the previous simulations.

We run each simulation for 2000 iterations, which is equivalent to 200 generations (if we take generation to mean a complete replacement of the population). For §4, we plot the posterior of the oldest agent in the population averaged over 100 simulation runs.

## 4. Results and discussion

We used our simulation model to test our two hypotheses in turn.

### (a) The trade-off hypothesis

**Figure 1.**
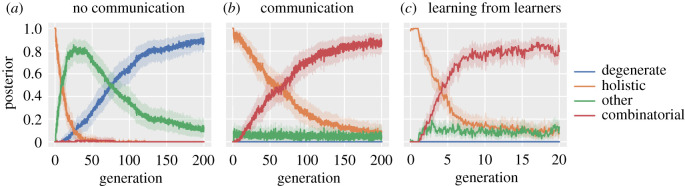
The posterior probability of each type of language in the oldest agent in the population averaged over 100 simulation runs. Panel (*a*) shows results with individuals learning from the oldest in the population, but without any communicative rationality. Panel (*b*) is the same but with communicative rationality. Panel (*c*) is the same as the second but with individuals learning from someone who themselves hasn’t finished learning. Graphs (*a*) and (*b*) represent 2000 iterations of the simulation, which is equivalent to 200 ‘generations’ (i.e. complete replacements of the population). *Note however, that part (c) shows only 200 iterations, equivalent to 20 generations*. Degenerate languages replace the initial holistic ones unless there is a pressure for communication, in which case combinatorial languages emerge eventually, supporting the **trade-off hypothesis**. The evolution of combinatorial languages is hugely accelerated if individuals who are still learning provide data for other learners, supporting the **learning from learners hypothesis**.

Figure 1*a* shows the result of running the simulation with a low communicative rationality parameter, *γ* = 0. This models a scenario where producers will use signals in proportion to their likelihood of being associated with the correct meaning but with no down-weighting of this preference based on how ambiguous those signals are. In other words, producers in this population care only about whether the signals have the correct meaning, but not about how functional the signals are in expressive/communicative terms. What we see here is the loss of the initial holistic language in favour of degenerate ones.^5^ In other words, the languages that emerge are ones that map all the four meanings to the same signal.

Why does this happen? What we are seeing here is a result familiar in models of iterated learning: convergence to the prior [5]. Over time, all other things being equal, behaviours transmitted by iterated learning will tend to converge on a stationary distribution in which different types of behaviour are represented with a frequency equal to their prior probability in learning.^6^ Since we are using a simplicity prior, it makes sense that the simplest languages (the ones in which every meaning is expressed by a single signal) will dominate. To put it another way, the easiest languages to learn in the set of possible languages are the degenerate ones, and since the only pressure on the languages being transmitted is that they be learned each generation, the tendency will be for languages to adapt to be more learnable through cultural evolution.

Figure 1*b*, on the other hand, shows what happens when we increase the communicative rationality of individuals in the population (here *γ* = 100, meaning producers will avoid signals in languages that are ambiguous). Here, we again see the loss of the original holistic language, but now see the emergence of the systems that look like human languages, in that they exhibit combinatorial structure. Duality of patterning has evolved in this scenario.^7^

Unlike in the previous set of simulations, when there is a high communicative rationality parameter, degenerate languages are highly unstable because even if they were to have a high posterior probability, signals *other* than the one that was used in that degenerate language would nevertheless be preferred by producers. This means that the language that the next generation would be exposed to would not look like a degenerate one, and therefore the language they learn would be less likely to be degenerate (despite having a high probability in the prior).

The combinatorial languages are the simplest languages (i.e. the languages with the highest prior probability) that nevertheless exhibit no ambiguity. As such they offer an optimal trade-off between the pressure for learnable languages that cultural transmission creates, and the pressure for expressive languages that rational communication creates. Degenerate languages are the most learnable due to their simplicity, but they are inexpressive. On the other hand, both holistic languages and combinatorial languages are maximally expressive, but of these two types holistic ones are the harder to learn.

In this sense, just as we have seen for other aspects of linguistic structure, duality of patterning can be explained in terms of an optimal trade-off between learning and communication. This trade-off emerges naturally from a process of cultural evolution among agents who are learning their language from previous generations, and using it in a way that is communicatively rational.

### (b) The learning from learners hypothesis

Figure 1*c* shows the results of running this simulation with communicatively rational agents (*γ* = 100, as before). The results are dramatically different. Note the different timescale here. After only four generations, combinatorial languages overtake the original holistic languages in the majority of the runs of the simulation.

These results demonstrate that just changing who learners are learning from can accelerate the evolution of structure in a language by more than an order of magnitude even when every other aspect of the simulation is held constant.^8^ We suggest that this provides support for the learning from learners hypothesis. Because deaf community languages involve contexts where there is substantial peer-to-peer interaction *in the process of language formation*, these are the languages where we expect to see more evidence for duality of patterning earlier in their history. Note that this does not mean that we necessarily expect to see lots of minimal pairs in the lexicon of a young deaf community sign language straight away. This is still a process of cumulative cultural evolution. Nor do we predict a difference in the long run between these two scenarios for language emergence. In both sets of simulations, combinatorial structure wins out in the end. However, these results do suggest that a consideration of who is learning from whom can radically change our predictions about the relative rate of cumulative cultural evolution of linguistic structure.

Why might this be? Over the lifetime of a learner, the influence of their prior reduces relative to the data that they are exposed to. In the model, this is a straightforward consequence of Bayes' rule. With no data, the posterior is equal to the prior. With one piece of data, the posterior is the product of the likelihood of that piece of data and the prior. With each subsequent piece of data, the posterior is updated by multiplying it with the likelihood of that data. As a result, the influence of the prior can be overwhelmed by data over time. Since the behaviour of agents is determined by their posterior (plus their communicative rationality parameter) then less experienced learners will inevitably tend to produce data that are more reflective of their prior.

This suggests that, as a straightforward consequence of the way learning works, less experienced learners are more likely to alter the language they are exposed to such that it fits their general biases favouring simplicity. However, as they receive more data about the nature of the language around them, they may ‘retreat’ from any simplifying generalizations in the light of evidence for complexity in the data. In our model, this is not due to some externally driven maturational change in the learners, but purely as a consequence of the relative weighting of prior bias and lived experience.

Crucially, for the cultural evolution of language, the data that a learner learns from are themselves the product of learning. If learners are exposed to a sufficient amount of data produced by individuals who themselves haven’t been exposed to all the data that they will be exposed to in their lives, then they are being given data upon which the prior bias for simplicity has had a greater influence.

The literature on the cultural evolution of language has identified the *bottleneck* on cultural transmission to be an important driver of the evolution of structure [9]. The idea is that if there are insufficient data for one generation of learners to acquire a language with irregular structure, then of necessity the language will need to adapt so that it can be learned from whatever amount of data that learners will be exposed to. To put it another way, to survive from generation to generation, language has to repeatedly squeeze through the narrow bottleneck of the finite data that a learner is exposed to. If this bottleneck is wide enough, then unstructured, complex language can get through, but when the bottleneck is narrow, structured, simpler languages that can be generalized from a small sample of data are the only ones that are stable from one generation to the next.

Our results here suggest a slight alteration to this view of the bottleneck on transmission. In both of our sets of simulations, learners are exposed to exactly the same amount of data, but in the second set of simulations it is as if the bottleneck were tighter. We argue that it is not the total amount of data that is important, but how much data a learner has been exposed to *at the point where they themselves contribute to the next generation’s input*. Children producing linguistic behaviour that is simpler (i.e. more systematic) than their input don’t typically get to influence the language of the next generation. By the time they are at an age where they have significant influence on the language of their linguistic community, the data that they have been exposed to may have overwhelmed the bias for simplicity. As a result, the process of simplification and systematization is slow. However, it is exactly in scenarios like the emergence of a deaf community sign language where we can expect that the influence of learners will shape the cultural transmission of the evolving language, causing rapid evolution of linguistic structure as a result.

## 5. General discussion

Our simulation results lend support to the idea that the cultural evolution of language gives rise to the kind of structure we see as typifying human language (its design features). It does this by mediating between two forces that act on an evolving language:

— Simplicity, arising from the prior bias of language learners.
— Expressivity, arising from the communicative rationality of language users.

As with other features that have been investigated in this framework in the past [59,63,64], combinatorial structure arises as an optimal trade-off between these two. Combinatorial languages are expressive in that they can be used to discriminate between different meanings, but they are also relatively simple in that their signals involve the reuse of a smaller set of elements in different combinations.

Our results also indicate a role for population structure in changing the rate at which an initially unstructured language will change into a combinatorially structured one exhibiting duality of patterning. When learners learn from other learners, this process is accelerated compared to when learners only learn from more experienced individuals. In other words, languages reflect the population context in which they emerge, as predicted by the Linguistic Niche Hypothesis [23].

It is important to note that combinatoriality is not necessary for a language to support successful communicative function. In our simulation, ABSL would be modelled as a holistic language, made up of a set of distinct signs that do not appear to have internal structure of recombinable meaningless sub-units. As such, it is just as capable of conveying meaning without ambiguity as combinatorial languages.

Sign language emergence seems to be shaped by the specific social context in which it happens. In the social contexts in which languages such as ISL emerged, new learners’ simplicity prior bias plays a determining role. The initial communicative signals may be contributed by the children’s *homesigns*, which are structurally simple systems that develop spontaneously in deaf children of hearing parents who are raised without accessible input [72,73]. Homesign systems show some combinatoriality [74]. This initially surprising feature lends support to our assumption of *a prior* bias for simplicity in language learners: without linguistic input, homesigners produce simple output *de novo*. While each child’s homesign may exhibit combinatoriality, the aggregate output of all children in the school setting would not, because the combined elements would be unique to each child and would not repeat across children.^9^ When the first groups of learners join the school community, they start to negotiate new conventional shared signs. During this process, their simplicity prior bias leads them to detect elements such as handshapes and locations that are reused across signs and to reuse them further, thus tending to make the system more combinatorial. Incoming new learners will learn from fellow learners whose output is systematic in this way. Thus, only a few new cohorts of learners are sufficient for simplicity to cumulatively evolve in the language. By contrast, in the village setting, although new learners may receive some input horizontally from other simplicity-driving learners, their input may be predominantly vertical, from older individuals who have had the time to successfully learn the non-systematic forms in the language. Overall, the linguistic input to learners is therefore more complex in the village than in the school.

Our findings may be cautiously extrapolated to the emergence of language in our species. The school setting described above, with its high-frequency horizontal transmission among young learners, represents an unusual social arrangement. Under normal circumstances, language is transmitted mostly vertically and obliquely, from older to younger individuals; horizontal transmission is attested between adults [75], but it is rare among very young learners. When a new communication system is created, for instance among speakers of different languages, the initial elements tend to be idiosyncratic, unstructured signals rooted in the context of interactions among small numbers of users [76–78]) and as such they tend to be holistic and unstructured. If language first emerged in the context of transmission from adults to children, these signals would be initially complex and it is likely that combinatoriality evolved slowly. (This proposal, however, does not take into account coevolutionary processes whereby exposure to protolinguistic behaviour prompted the biological evolution of new social–cognitive adaptations that, in turn, changed the nature of the protolinguistic behaviour [79,80].)

Finally, our results for the rate of evolution of combinatoriality beg the question of whether other design features of language such as compositionality and arbitrariness—and indeed the levels of complexity of cultural traits beyond language, for instance in technology, social interaction or art—are also affected by patterns of transmission modulating the effects of cognitive biases. This can be tested in future simulations and experiments.

## 6. Conclusion

Language can fulfil its function as repository and mediator of collective knowledge thanks to design features such as duality of patterning. Our work has shown that combinatoriality, the most fundamental aspect of duality of patterning, emerges in language thanks to the same processes as another aspect, compositionality. It is a trade-off between the cumulative evolution of expressivity as an adaptation to communication, and the cumulative evolution of systematic structure as an adaptation to a cognitive learning prior bias for simplicity.

Furthermore, our results suggest that interaction dynamic patterns in the social network affect the rate of evolution of combinatoriality because they modulate the interactions between cognitive biases and data. This is a possible explanation for the unique and puzzling absence of this feature of language in ABSL, an emergent sign language that developed in a village setting (arguably like most languages did, spoken or signed) where learners tend to learn from mature producers. In other signed languages that developed in school settings, with a high prevalence of learning from other learners, combinatoriality may have emerged at an unusually rapid pace.

Overall our work indicates that a few general cognitive and functional pressures (for learnability, efficiency and effectiveness) shape the structure of cultural traits, and that the rate of cultural evolution is modulated by properties of the context in which cultural evolution occurs, in particular by population structure and dynamics.

## Footnotes

1 As Ladd [46] points out, it is not clear that compositionality is actually required for Hockett’s conception of duality of patterning. What is clearly required is for there to be a layer of structure where there are signals that carry meaning, and a second layer where signal elements that themselves do not carry meaning are recombined. So, for example, an animal signalling system—akin to a language with only one-word sentences—in which different signals had different meanings, and those signals were made up of recombinations of parts could be considered to exhibit duality of patterning even if there were no compositional recombination to create more complex meanings out of smaller meaningful units. This is why we focus on *combinatoriality* in this paper.

2 The code for the model described here, complete with the necessary parameters to recreate all the graphs in this paper, is available as a Jupyter notebook here: https://github.com/smkirby/combinatoriality.

3 Recall, these ‘characters’ are really stand-ins for whatever the atomic elements are in the model—such as handshape and orientation. In this sense, the model presupposes that there is *some* level of analysis where such atomic elements lie, and that these can be smaller than the whole word. This is a significant simplification, which assumes that there exists a learning process that can take extract such features from continuous signals. The work by Hofer and colleagues is a promising direction for uncovering this learning process [53]. However, it is important to note—as we will see—that this assumption does not build in the result that combinatorial systems are inevitable.

4 Note that this process of production, biased as it is by communicative potential, means that the actual process of production is not the one that learners of the language assume is taking place when calculating the posterior probability of each language. In other words, *C*(*s*|*l*, *m*) is not necessarily proportional to the likelihood term *P*(*s*|*l*, *m*). This means that our model of an agent strictly speaking deviates from that of a pure rational learner.

5 The initial rise in the ‘other’ language type here is due to the initial holistic language gradually giving way to completely degenerate ones via a language that is partially holistic and partially degenerate.

6 Strictly speaking, the convergence to the prior result does not necessarily hold in this case, as agents are learning from multiple other agents in their lifespan, and the agents are not rational learners in the sense that the likelihood they use to calculate the posterior probability does not take into account the fact that they are learning from what are potentially multiple other languages [70,71].

7 Note that here we don’t see a rise in the ‘other’ language types, because the communicative rationality parameter strongly disfavours languages with even partial ambiguity.

8 We also ran the simulation using agents with *γ* = 0 but learning from learners, and saw that the convergence to degenerate languages was accelerated in this case as well.

9 The simulations we report above take as their starting point a shared holistic language. However, if we start instead with a population of distinct combinatorial homesign-like systems, we do indeed see precisely this. First, a shared holistic language appears, followed only later by the reemergence of a combinatorial language. For more information, see the Jupyter notebook associated with the paper here: https://github.com/smkirby/combinatoriality.

## Data accessibility

The code for the model described in the paper, complete with the necessary parameters to recreate all the graphs, is available as a Jupyter notebook here: https://github.com/smkirby/combinatoriality.
